# Identification of germline cancer predisposition variants during clinical ctDNA testing

**DOI:** 10.1038/s41598-021-93084-0

**Published:** 2021-07-01

**Authors:** Leigh Anne Stout, Nawal Kassem, Cynthia Hunter, Santosh Philips, Milan Radovich, Bryan P. Schneider

**Affiliations:** 1 Indiana University School of Medicine, 1030 W. Michigan St., Suite 3307, Indianapolis, IN 46202 USA; 2 Indiana University Health Precision Genomics, Indianapolis, IN USA

**Keywords:** Cancer genetics, Cancer genomics, Mutation

## Abstract

Next-generation sequencing of circulating tumor DNA (ctDNA) is a non-invasive method to guide therapy selection for cancer patients. ctDNA variant allele frequency (VAF) is commonly reported and may aid in discerning whether a variant is germline or somatic. We report on the fidelity of VAF in ctDNA as a predictor for germline variant carriage. Two patient cohorts were studied. Cohort 1 included patients with known germline variants. Cohort 2 included patients with any variant detected by the ctDNA assay with VAF of 40–60%. In cohort 1, 36 of 91 (40%) known germline variants were identified through ctDNA analysis with a VAF of 39–87.6%. In cohort 2, 111 of 160 (69%) variants identified by ctDNA analysis with a VAF between 40 and 60% were found to be germline. Therefore, variants with a VAF between 40 and 60% should induce suspicion for germline status but should not be used as a replacement for germline testing.

## Introduction

Tumor next generation sequencing (NGS) has become commonplace in routine clinical practice for patients with a variety of malignancies to help identify potential drug targets. We, and others, have demonstrated that in the process of tumor NGS approximately 3–13% of patients are found to harbor incidental germline pathogenic variants^1–7^. While not the goal of tumor NGS, these findings can have major implications for healthy family members^8^. While traditionally established risk factors (e.g. age of diagnosis, family history, and tumor characteristics) have prompted the consideration for germline testing^9^, more recently, expert consensus guidelines have also considered specific findings on tumor NGS. The current NCCN guidelines recommend referral for appropriate germline genetic testing any time a variant is identified through tumor NGS that would have clinical implications if the variant was determined to be pathogenic and germline in origin^10^.

While reacting to findings on tumor NGS has clearly augmented the identification of confirmed pathogenic germline variants, tumor NGS should not be considered a substitute for germline testing. Tumor NGS is designed to identify drug targets which has implications for both the variant coverage and the definition of pathogenicity. Specifically, tumor NGS tests are designed to uncover variants that would have therapeutic implications and these variants are not in complete concordance to those that would confer risk of disease^11^. Second, the evidence to define whether a variant can impact drug responsiveness is different from that required to prove it increases the risk of disease; thus leading to an incongruence in the determination of pathogenicity between a tumor NGS vendor and ClinVar (or other high-quality databases used to determine germline pathogenicity). Further, it is important to note that some pathogenic germline variants are missed by tumor panels due to the variant type including structural rearrangements and/or a variant being present in a pseudogene region^12,13^.

Recently, plasma-based assessment for mutations in ctDNA has become a non-invasive and relatively quick way to assess for tumor specific mutations^14^. Prior studies have demonstrated high, although not perfect, concordance of variants detected in ctDNA with matched tumor^15,16^. Unlike tumor-based NGS assays, commercial plasma ctDNA tests commonly report the variant allelic frequency (VAF). Inclusion of VAF can be used to assess for clonal vs. subclonal mutations or heterogeneity. In addition, the VAF might also have implications for its origin; with VAF’s near 50% suggesting a possible germline variant. In this manuscript, we report the concordance between the VAF from ctDNA analysis and germline carrier status utilizing two patient cohorts sequenced at the Indiana University Health Precision Genomics program. Cohort 1 included patients known to carry a germline pathogenic variant(s) with matching ctDNA assessment available for comparison. Cohort 2 included patients identified to carry a variant in ctDNA with a VAF between 40 and 60% with germline variant status available for comparison.

## Methods

### Identification of germline variant carriers

This study was approved by the Indiana University Institutional Review Board (IRB). All research was conducted in accordance with the WMA Declaration of Helsinki. Patients who were part of the IU Health Precision Genomics Program who had evidence of a pathogenic germline variant and who also had ctDNA tumor assessment were considered. Germline analysis was completed by NantOmics and/or commercially available CLIA-certified laboratories on either blood or saliva samples. Germline analysis performed by NantOmics included whole exome sequencing (WES) of germline DNA with CLIA reporting of the ACMG cancer predisposition genes. DNA sequencing libraries were prepared from normal blood or buccal samples using the KAPA Hyper prep kit and sequenced on an Illumina Sequencing Platform. DNA sequencing data was aligned to the human genome (hg19) using the bwa algorithm. Duplicated reads were marked by samblaster, and indel realignment and base quality recalibration was performed using GATK v2.3. Each variant was sequenced to a minimum depth of 10 reads and had a minimum alternate allele fraction of 0.25 in the normal sequencing data. VCF files containing germline variants were generated. The NantOmics WES CLIA-sequencing has demonstrated > 95% sensitivity and > 99% specificity for germline SNPs and germline insertions and deletions. Other germline variant carriers were identified by CLIA-vendors including: Ambry, GeneDx, Invitae, and Myriad. Those with a variant classified as pathogenic or likely pathogenic were considered to have a pathogenic variant. All others were considered to not have a pathogenic variant.

### Assessment of ctDNA

Comprehensive genomic profiling was performed on hybridization-captured, adaptor ligation-based libraries of 62 (all coding exons of 27 genes, select exons of 34 genes, FoundationACT) or 70 (all coding exons of 35 genes, select exons of 35 genes, FoundationOne Liquid) cancer-related genes plus selected introns from 6 (FoundationACT) or 7 (FoundationOne Liquid) genes frequently rearranged in cancer to identify base substitutions, small insertions or deletions, focal copy number alterations (amplifications), and rearrangements, as previously described^17^. Plasma based liquid biopsy testing was performed in a CLIA–certified, College of American Pathologists–accredited reference laboratory (Foundation Medicine, Cambridge, MA).

### Cohort 1: Identification of matching variants in ctDNA in patients with a known germline variant

For patients with a known germline variant, this same variant was queried for in the ctDNA results. VAF of the ctDNA was not considered. Correlation between the candidate germline variant and the presence or absence in the ctDNA was calculated as a percentage of concordance.

### Cohort 2: Assessment of concordance between variants with VAF of 40–60% on ctDNA and germline carriage

Patients who were part of the IU Health Precision Genomics Program who germline data (as defined above in cohort 1) with ctDNA tumor assessment and a candidate VAF of 40–60% within the following cancer predisposition genes were considered: *ALK, APC, ATM, BRCA1, BRCA2, CDH1, CDK4, CDKN2A, CHEK2, KIT, MET, NF1, PALB2, PDGFRA, PTEN, RB1, RET, STK11*, and *TP53* (n = 111). Concordance between the carriage of a candidate variant with VAF of 40–60% with germline carriage was calculated as a percentage of concordance.

### Variant interpretation for pathogenicity

All variants with an allele frequency of 40–60% regardless of pathogenicity were included in this analysis. Somatic variants identified by ctDNA analysis were interpreted as “pathogenic” if the variant was listed in the main section of the report (i.e. not in the variant of unknown significance appendix). Pathogenicity of germline variants was determined using the overall interpretation conferred in ClinVar. Variants with conflicting interpretations of pathogenicity in ClinVar were manually reviewed by a licensed genetic counselor to determine pathogenicity. All pathogenic and likely pathogenic variants were classified as “pathogenic”. All other variants, including those that were not listed in ClinVar, were classified as “not pathogenic”.

## Results

### Cohort 1: Frequency of germline mutations detected in ctDNA

**Figure 1:**
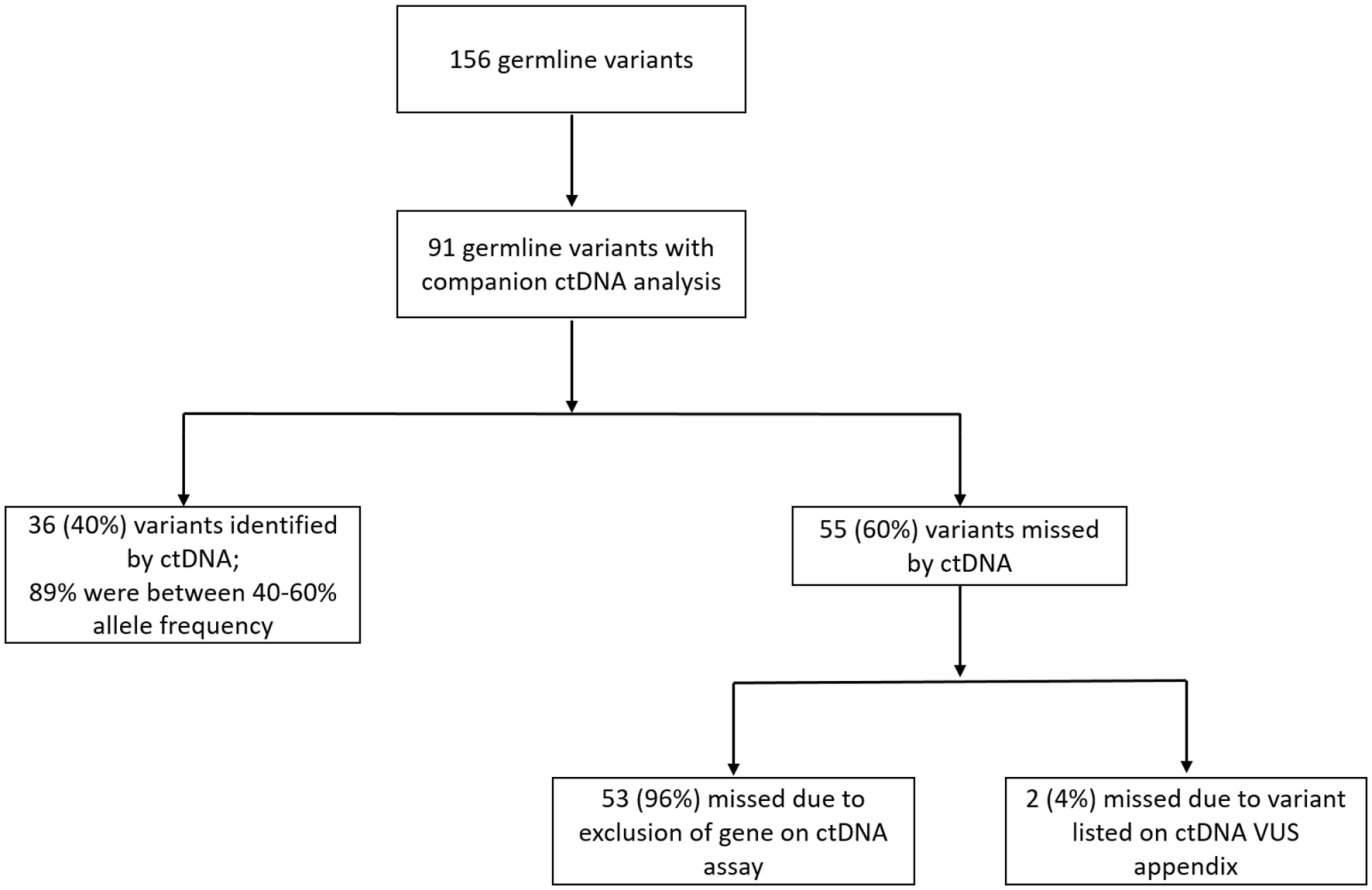
CONSORT Diagram for cohort 1.

Of patients seen at the IU Health Precision Genomics Program, a total of 156 pathogenic germline variants were identified (CONSORT diagram 1). ctDNA results using FoundationOne Liquid were available for 86 patients (91 variants). Demographics for these 86 patients are summarized in Table 1. Thirty-six of the 91 variants (39.5%) were identified by ctDNA analysis. The results of Cohort 1 analysis are summarized in Fig. 1. The most common mutations were in *BRCA2* (n = 10) and *BRCA1* (n = 8) (Table 2). The average VAF for germline mutations detected in ctDNA was 52.1% with a median of 50.3% and a range of 39% to 87.6%. Of the germline variants identified, 88.9% had a VAF within the range of 40–60%.

**Table 1:** Cohort 1: Patient characteristics and tumor types for known germline carriers with companion ctDNA analysis (n = 86).

Characteristics	No. (%)
**Age**	
< 45	18 (21)
46–60	35 (41)
61–75	30 (35)
> 76	3 (3)
**Sex**	
Female	46 (53)
Male	40 (47)
**Race**	
White/Caucasian	79 (92)
Black/African American	5 (6)
Unknown	2 (2)
**Tumor types**	
Breast	17 (20)
Sarcoma	11 (13)
Colorectal	10 (12)
Ovarian	7 (8)
Prostate	7 (8)
Lung	5 (6)
Cholangiocarcinoma	4 (5)
Esophageal	4 (5)
Other	21 (24)

**Table 2:** Cohort 1: Breakdown of germline mutations missed or identified using ctDNA analysis.

Gene	Missed	Identified
	No. of mutations	No. of mutations
*BRCA2*	0	10
*BRCA1*	0	8
CHEK2*	10	5
*CDKN2A*	2	4
*TP53*	0	3
ATM*	8	2
*NF1*	0	2
*KIT*	0	1
*RET*	0	1
*APC†*	1	0
*MLH1†*	1	0
*MITF†*	7	0
*SDHA†*	1	0
*NBN†*	3	0
*RECQL4†*	1	0
*FANCC†*	3	0
*NTHL1†*	5	0
*PALB2†*	3	0
*FLCN†*	1	0
*RAD51D†*	1	0
*HOXB13†*	3	0
*BRIP1†*	2	0
*MSH2†*	1	0
*BMPR1A†*	1	0
*PMS2†*	1	0
Total	55	36

*Gene added to ctDNA assay after start of study.

^†^Gene was not included on ctDNA assay at the time of testing.

Conversely, 55 of 91 known germline variants (60.4%) were not identified by ctDNA analysis. The most commonly missed variants were in *CHEK2* (n = 10) followed by *ATM* (n = 8); (Table 2). Notably, all variants not identified by ctDNA were not identified due to the ctDNA assay’s exclusion of the gene or due to discrepant interpretations of pathogenicity between the somatic and germline labs. Of note, at the initiation of this analysis, the *CHEK2* and *ATM* genes were not included in the ctDNA assay but have subsequently been added to the assay. Germline variants in the *CHEK2* and *ATM* genes were identified by ctDNA analysis 100% of the time if the gene was included in the ctDNA assay. Fifty-three of 55 (96%) missed variants were missed due to exclusion of the gene by ctDNA assay. Three of these variants were in 3 patients with germline mutations in Lynch syndrome associated genes. Two of these patients had MSI-high tumors while the third patient had a MSI-stable tumor. Two of 55 variants (4%) not detected by ctDNA analysis were considered missed variants because they were listed on the variants of unknown significance appendix of the ctDNA report.

### Cohort 2: Likelihood of a ctDNA variant with an allele frequency of 40–60% representing a germline mutation

**Figure 2:**
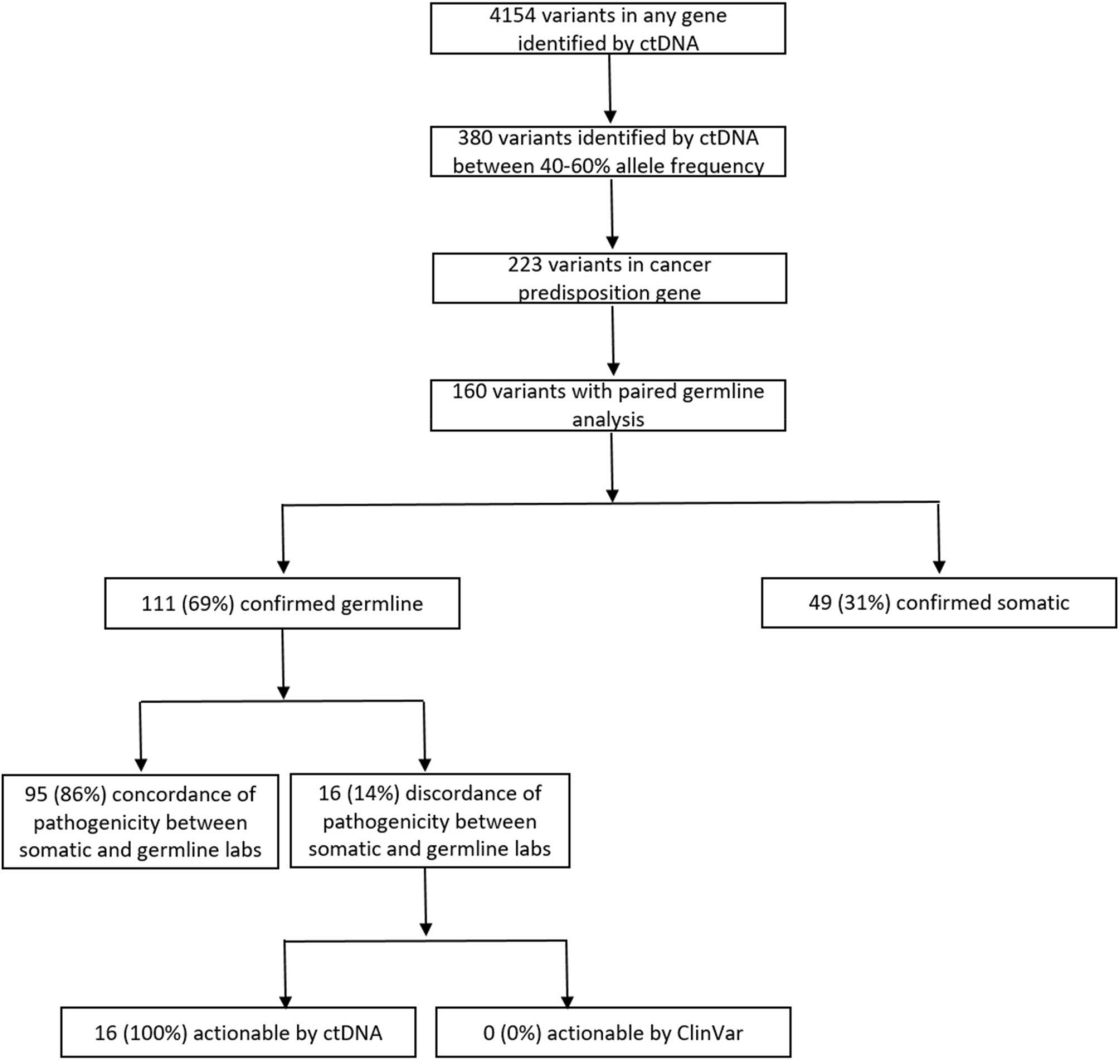
CONSORT Diagram for cohort 2.

**Figure 3:**
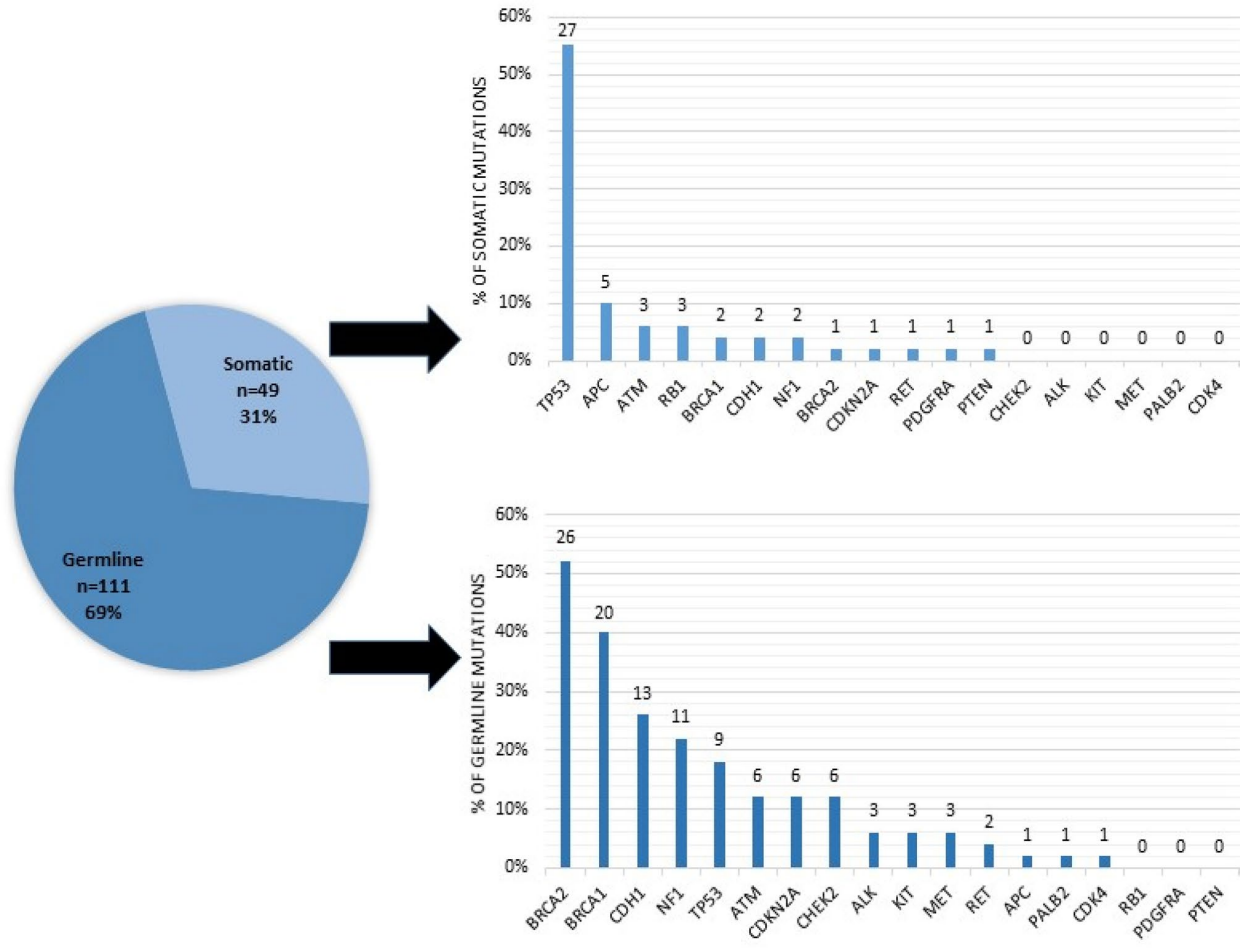
Distribution of germline and somatic mutations in hereditary cancer genes between 40 and 60% allele frequency identified by ctDNA analysis. *The number above each bar represents total number of cases.

A total of 4,154 variants were identified in 1,255 patients that underwent ctDNA NGS through the IU Health Precision Genomics Program (CONSORT diagram 2). 130 patients (n = 160 variants) had an evaluable candidate germline variant(s) (Table 3) with an allele frequency between 40 and 60%; irrespective of pathogenicity on ctDNA analysis. One hundred and eleven of 160 (69.4%) variants were found to be germline in origin and 49 of 160 (30.6%) were found to be somatic in origin. The results of Cohort 2 analysis are summarized in Fig. 2. The most common germline variants identified were in *BRCA2* (n = 26), *BRCA1* (n = 20), *CDH1* (n = 13), and *NF1* (n = 11) (See Table 4 for full list). Only *TP53* and *APC* were more likely to be somatic in origin (< 50% germline) when the allele frequency in the ctDNA was between 40 and 60% (considering genes where there were at least 5 cases). Specifically, *TP53* variants (n = 36) were germline in 25% of cases and *APC* variants (n = 6) were germline in 16.7% of cases. The distribution of germline and somatic mutations in hereditary cancer genes between 40 and 60% allele frequency identified by ctDNA analysis are depicted in Fig. 3.

**Table 3:** Cohort 2: Patient characteristics and tumor types of patients with a variant identified between 40 and 60% allele frequency in a cancer predisposition gene on ctDNA analysis (n = 130).

Characteristics	No. (%)
**Age**	
< 45	25 (19)
46–60	56 (43)
61–75	45 (35)
> 76	4 (3)
**Sex**	
Female	73 (56)
Male	57 (44)
**Race**	
White/Caucasian	111 (85)
Black/African American	12 (9)
Unknown	3 (2)
Asian	3 (2)
American Indian	1 (1)
**Tumor types**	
Breast	23 (18)
Colorectal	19 (15)
Sarcoma	16 (12)
Ovarian	11 (8)
Lung	10 (8)
Prostate	9 (7)
Pancreas	9 (7)
Head and neck	5 (4)
Other	28 (22)

**Table 4:** Cohort 2: Likelihood of ctDNA mutation between 40 and 60% allele frequency being germline in origin.

Gene	No. of mutations	No. germline (%)
*TP53*	36	9 (25)
*BRCA2*	27	26 (96.3)
*BRCA1*	22	20 (90.9)
*CDH1*	15	13 (86.7)
*NF1*	13	11 (84.6)
*ATM*	9	6 (66.7)
*CDKN2A*	7	6 (85.7)
*CHEK2*	6	6 (100)
*APC*	6	1 (16.7)
*ALK*	3	3 (100)
*KIT*	3	3 (100)
*MET*	3	3 (100)
*RET*	3	2 (66.7)
*RB1*	3	0 (0)
*PALB2*	1	1 (100)
*CDK4*	1	1 (100)
*PDGFRA*	1	0 (0)
*PTEN*	1	0 (0)
Total	160	69.4 (111)

### Concordance of pathogenicity between germline and somatic labs

Of 111 germline variants between 40 and 60% allele frequency on ctDNA analysis, 95 variants (86%) had concordance of pathogenicity between the germline (as determined by ClinVar) and the somatic lab (as determined by the ctDNA CLIA front page report). Of the 16 variants (14%) with discordance of pathogenicity, 100% were interpreted as pathogenic by the somatic testing lab and 0% were interpreted as pathogenic in the germline.

## Discussion

Plasma ctDNA NGS is a commonly employed test designed to identify drug targets for patients with advanced cancer. ctDNA NGS has several advantages over tumor-based NGS including its ability to capture molecular heterogeneity and its ability to be ascertained in a non-invasive manner. While not designed to identify germline variants, our data demonstrates that the commonly reported plasma ctDNA VAF also sheds substantial insight into the origin of the variant; germline or somatic.

Herein we report concordance of germline variants with variants identified through plasma ctDNA NGS in patients with advanced cancer as part of the Indiana University Health Precision Genomics Program where the goal of NGS was to uncover drug targets. In cohort 1, we evaluated a group of patients who were known to carry a pathogenic germline variant. When the germline variant was identified by ctDNA assessment, we found 88.9% of the variants had a VAF between 40 and 60%; with the lowest VAF reported at 39%. In cohort 2, 69% of variants in a cancer predisposition gene with a VAF of 40–60% were confirmed to be germline. These findings show striking concordance between germline variation and its reflection in the plasma ctDNA.

While these data support that the identification of a pathogenic variant on ctDNA with a VAF of 40–60% should be considered for confirmatory germline testing, this should not be considered an acceptable screening technology for germline testing. Importantly, coverage for pathogenic germline variants is not comprehensive and the concordance was high only if the appropriate gene and variant was considered. Specifically, 60% of our known carriers of a germline pathogenic variant did not have their respective germline variant identified at any percentage concentration by plasma ctDNA analysis. In most cases this was the result of the involved gene not being incorporated into the ctDNA assay. Importantly, three patients known to have Lynch syndrome did not have their respective Lynch syndrome-associated pathogenic variant identified on their ctDNA analysis. In our dataset, a total of 7 variants (5 in *CHEK2* and 2 in *ATM*) were also considered missed variants because the respective genes were not analyzed by the assay. Demonstrating the evolution of these assays, however, the *CHEK2* and *ATM* genes have now been subsequently added to the ctDNA analysis and these variants would now be detected by the assay. Additionally, while the majority of patients with the germline variant identified in the plasma had a VAF between 40 and 60%, over 10% of variants were outside of this range and would have been missed should a tight threshold around 50% had been applied as a screen.

Conversely, not all patients that carry a predisposition variant with a VAF near 50% had a germline mutation. VAF’s approaching 50% in *TP53* and *APC* would raise concerns for the possibility of Li-Fraumeni syndrome and Familial Adenomatous Polyposis, respectively. We found that variants in *TP53* and *APC* with a VAF 40–60%, however, were markedly more likely to be somatic in origin as opposed to germline. Only 25% (9 of 36 cases) of *TP53* variants and 17% (1 of 6 cases) of *APC* variants were confirmed to be germline in origin. Even with low likelihood of hereditability, the clinical implication of carriage, however, do not allow for dismissal when identified. Thus, for these genes, clinical judgment of the patient’s personal and family history remains important in the effort to identify patients with an underlying hereditary risk factor to determine the benefit from confirmatory germline testing.

Finally, the definition of pathogenicity between a germline variant and that reported by the somatic vendor report are not 100% concordant and are dynamic. As the data evolve for drug target predictability, a gene/variant previously not defined as actionable may change as was seen with the *CHEK2* (n = 15) and *ATM* carriers (n = 10) in our dataset. We also found that the somatic vendors were more liberal with defining a variant as pathogenic (for drug target) than ClinVar (for disease risk). Regardless, the lack of concordance should serve as a reminder that the definition criteria for drug sensitivity and risk are different and can result in missing a germline variant when only interpreting the ctDNA results. This difference in definition was critical in 55 cases from this dataset; having been listed as a variant of unknown significance on the vendor’s interpretation sheet (2 cases) or completely missing in the rest.

Traditionally, patients have been considered for genetic testing based on well-established criteria including age at diagnosis, family history, and tumor characteristics. This approach is far from perfect and improvements in identifying patients with an underlying hereditary risk factor are needed and are being uncovered. While not the intent of plasma ctDNA NGS, findings from this test can provide additional insights and guidance to direct the consideration of testing which in turn will allow for improvements in cancer control efforts. Equally important is the recognition that a “negative” ctDNA test should not be used to exclude patients from germline testing when clinically indicated. The resultant increase in uncovering an incidental germline variant through plasma ctDNA requires careful consideration of proper pretest counseling to allow for a patient to opt out of testing and to optimize the impact of any findings for that patient’s care. While these unexpected advances have led to additional need for counseling and expertise in interpretation, with thoughtful management prior to and after the test, many patients (and their relatives) might gain access to critical, and potentially life-saving, information.
